# *Aster* × *chusanensis* Growth and Phenolic Acid Composition under Different Cultivation Temperatures

**DOI:** 10.3390/plants13131855

**Published:** 2024-07-05

**Authors:** Han-Sol Sim, Hyuk Joon Kwon, Seong-Nam Jang, Ga Oun Lee, In-Je Kang, Gyu-Sik Yang, Gi-Heum Nam, Ji Eun Park, Ha Yeon Byun, Young-Hyun You, Ki-Ho Son

**Affiliations:** 1 Department of GreenBio Science, Gyeongsang National University, Jinju 52725, Republic of Korea; 2022213021@gnu.ac.kr (H.-S.S.); 220415251@gnu.ac.kr (S.-N.J.); 2023215175@gnu.ac.kr (G.O.L.); 2 Biological Resources Assessment Division, National Institute of Biological Resources, Miryang 50452, Republic of Korea; hyukjoon@kolmarbnh.co.kr (H.J.K.); namgih@korea.kr (G.-H.N.); pje12@korea.kr (J.E.P.); bh0484@korea.kr (H.Y.B.); 3 Division of Horticultural Science, College of Agriculture & Life Science, Gyeongsang National University, Jinju 52725, Republic of Korea; 2023210150@gnu.ac.kr (I.-J.K.); 2023210154@gnu.ac.kr (G.-S.Y.); 4 Species Diversity Research Division, National Institute of Biological Resources, Incheon 22689, Republic of Korea

**Keywords:** *Aster* × *chusanensis*, Asteraceae, cultivation temperature, growth, HPLC, phenolic acid, propagation, secondary metabolites

## Abstract

Plants of the Asteraceae family have been cultivated worldwide for economic, medicinal, and ornamental purposes, including genera such as *Aster*, *Helianthus*, and *Cosmos*. Numerous studies examined their secondary metabolites; however, those of *Aster* × *chusanensis*, which is a natural hybrid species in South Korea, are unclear, and optimized propagation methods should be identified. We analyzed phenolic acid concentrations in each part of *Aster* × *chusanensis* through HPLC. Further, we investigated the growth characteristics and secondary metabolite concentrations under various growth temperatures using division propagation, followed by growing at 20, 25, and 30 °C in a growth chamber. Chlorogenic acid was the primary compound, which was particularly high in the leaves. The growth characteristics did not differ significantly between temperatures, and 30 °C was most efficient for phenolic acid biosynthesis. Our results provide valuable information on optimized propagation and secondary metabolite concentrations under different temperatures of *Aster* × *chusanensis*.

## 1. Introduction

Plants of the Asteraceae family are frequently cultivated worldwide because of their prominent economic, medicinal, and ornamental purposes [1]. This family comprises approximately 2000 genera and 23,000 species [2]. *Aster*, *Helianthus*, *Callistephus*, and *Cosmos* are well-known Asteraceae genera. The genus *Aster* comprises several species, including *A. glehnii* F. Schmidt, *A. ageratoides* Turcz., *A. oharai* Nakai, *A. spathulifolius* Maxim., and *Aster* × *chusanensis*. Among these, *A. ageratoides* Turcz. is a traditional medicinal plant that is rich in phenolic compounds and is frequently cultivated in the Republic of Korea [3]. *A. glehni* is the principal native vegetable found on Ulleung Island, Republic of Korea, and its leaves contain numerous terpenoids, quinic acid derivatives, and flavonoids [4]. *A. oharai* Nakai is mainly distributed in the eastern area of Korea, and its aerial parts have been used in traditional Korean medicine to treat asthma and diuresis [5]. *Aster* × *chusanensis* Y.S. Lim, Hyun, Y.D. Kim and H.C. Shin was discovered in Ulleungdo, Ulleung-gun, Gyeongsangbuk-do, Republic of Korea, [6] and is a natural hybrid species whose parents are *A. pseudoglehnii* and *A. oharai* Nakai [7].

Many Asteraceae species have biological and medicinal properties. For example, sunflower (*Helianthus annuus* L.) seeds, an oilseed crop, possess anti-oxidant activity [8]. The roots of *Inula helenium* contain various secondary metabolites, such as flavonoids, terpenes, and phenolic acids [9].

Phenolic compounds exhibit anti-oxidant effects [10] and anti-allergic [11] and anti-arthritic activities [12]. *A. glehni* leaves can exert anti-oxidant, anti-inflammatory [13], sedative, and anticonvulsant effects [14], and ethanol extract from *A. glehni* can be used to treat gout owing to a xanthine oxidase inhibitor [15].

Plants use various defense mechanisms in response to environmental stress, such as producing secondary metabolites, and flavonoids and terpenoids have been shown to accumulate under low-temperature stress [16]. Furthermore, plants can survive in extreme environments by stimulating transcription factors related to the biosynthesis of phenolic compounds, such as anthocyanins and carotenoids, which occurs under high-temperature stress [17].

Closed-type plant production systems, such as vertical farms, offer a controlled environment for year-round crop production. This allows for the precise regulation of light, temperature, and humidity, thereby enabling the manipulation of specific environmental factors [18]. In this context, it is important to explore the potential of plants in the Asteraceae family for various applications in the food industry, cosmetics, and medicine. Comprehensive research is needed to fully understand and characterize these species, including their cultivation methods, identification, and the development of dietary supplements or pharmaceutical-based products [19]. In addition, elucidating the effects of growth temperature on phenolic acid degradation will help to determine their role in the accumulation of each part. Various Asteraceae species have been well studied with regard to their functional compounds; however, the functional compounds of *Aster* × *chusanensis* are unknown because the species was described only recently. Molecular studies have been performed to elucidate the hybridization origin and morphology of *Aster* × *chusanensis* [7], whereas basic research on its secondary metabolites is currently scarce. Therefore, this study was conducted to confirm efficient cultivation methods and secondary metabolite enhancement methods for domestic native species within closed-type plant production systems by (1) investigating the distribution of phenolic acids in all *Aster* × *chusanensis* plant parts and (2) confirming the changing pattern of phenolic acid concentration in each part according to different growth temperature conditions.

## 2. Results and Discussion

### 2.1. Phenolic Acid Concentrations of Aster × chusanensis Parts

**Figure 1:**
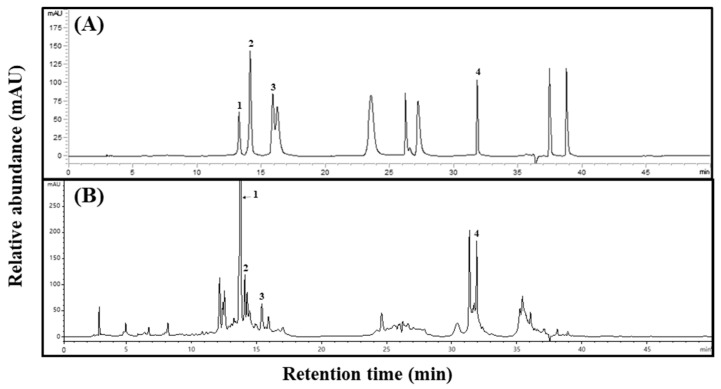
Phenolic acid HPLC chromatograms of *Aster* × *chusanensis* leaves. (**A**) Standard of phenolic acids and (**B**) leaves. The absorbance of individual compounds was detected at 280 nm. The concentration of the standards was 100 µg/mL, and the concentration of the extract was 50,000 µg/mL. Peak 1, chlorogenic acid; peak 2, p-hydroxybenzoic acid; peak 3, vanillic acid; peak 4, benzoic acid.

To confirm the specificity between standard and sample extracts, we confirmed the peak in the high-performance liquid chromatography (HPLC) chromatogram of *Aster* × *chusanensis* leaves and selected individual phenolic acids through matching retention times to the standards (Figure 1).

The phenolic acid concentrations differed between *Aster* × *chusanensis* parts (Table 1). The total phenolic acid concentration was highest (*p* < 0.01) in the leaves. In particular, chlorogenic acid showed a high concentration difference among plant parts, and the concentration in the leaves (28,215.82 µg g^−1^ DW) was approximately 24 times higher (*p* < 0.01) than that in the roots (1164.48 µg g^−1^ DW). A previous study reported that chlorogenic acid was the main phenolic compound in the seeds of sunflowers, which also belong to the Asteraceae family [20]. Chlorogenic acid can fulfil several therapeutic functions in the body, including neuroprotective, antiviral, antipyretic, free radical scavenging, and hepatoprotective effects [21]. p-hydroxybenzoic and benzoic acid levels were highest (*p* < 0.001) in the leaves. These two chemicals have antibacterial, antifungal, anti-inflammatory, and anti-oxidant properties and are used in drugs, cosmetic products, food, and beverages [22]. The leaves had the highest vanillic acid concentration, which can exert neuroprotective, immunostimulatory, and cardioprotective effects [23].

### 2.2. Growth Characteristic Analysis of Aster × chusanensis under Different Growth Temperatures

A closed-type plant production system and division propagation were used to study *Aster* × *chusanensis*. The division cultivation method is a form of plant propagation in which some plant parts are divided, and each part of the plant includes one or more root and stem parts. This method maintains species conservation and genetic diversity, and the cultivation period is shorter than that of seed propagation. Therefore, this method can improve crop productivity and target compound productivity by shortening the time to harvest [24]. Thus, this method is suitable for studying native species by obtaining many *Aster* × *chusanensis* individuals propagated in a short period of time.

**Figure 2:**
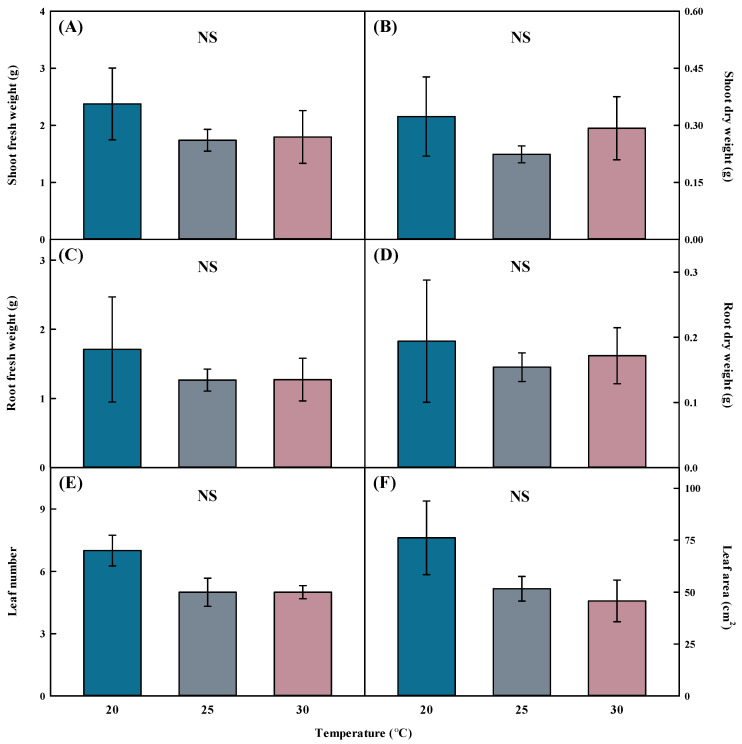
Shoot fresh weight (**A**), shoot dry weight (**B**), root fresh weight (**C**), root dry weight (**D**), leaf number (**E**), and leaf area (**F**) by different temperatures of *Aster* × *chusanensis* (*n* = 5). Bar heights indicate the mean, and error bars show the mean standard deviation. Mean separation by Duncan’s multiple range test at 5% level. NS—difference not significant.

No significant difference was observed in the fresh and dry weights of the shoots and roots when grown at 20, 25, and 30 °C on *Aster* × *chusanensis* transplanted using division cultivation (Figure 2). Growth temperature did not result in statistically significant differences in leaf number and area. Because *Aster* × *chusanensis* is a perennial plant that flowers from September to October [25], general growth is possible in a temperature range of 20–30 °C without growth deterioration, according to the results of this study. Therefore, no significant effects of temperature on growth characteristics were observed. At 20 and 30 °C, the standard error was higher than at 25 °C, confirming that uniformity was low in division seedling production, and 25 °C was the optimal temperature for producing uniform division seedlings. However, although the standard error was large at 20 °C, the values of all growth characteristics were high; therefore, 20 °C was the most efficient growth temperature for uniform seedling production.

### 2.3. Phenolic Acid Concentrations in Aster × chusanensis Shoots under Different Growth Temperatures

**Figure 3:**
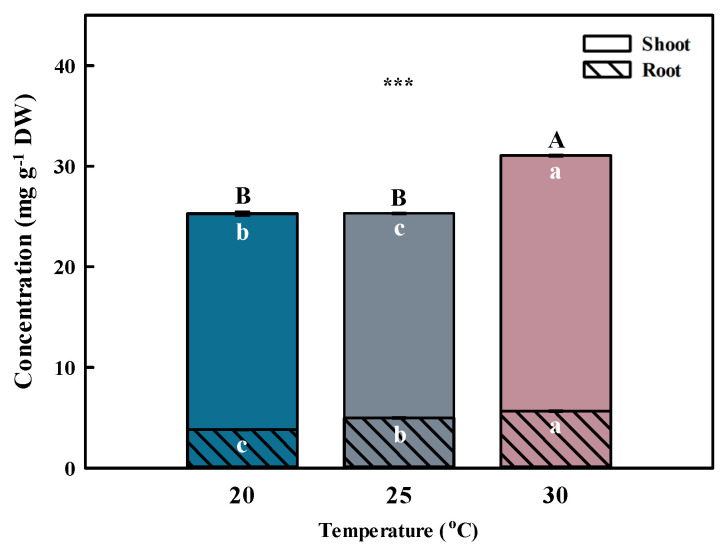
Total phenolic acid concentrations by different temperatures for the shoot and root of *Aster* × *chusanensis* (*n* = 8). Bar height indicates the mean, and error bars show the standard deviation. Mean separation by Duncan’s multiple range test at 5% level. Different letters (A–B, a–c) indicate significant differences at *p* < 0.001 (***).

**Figure 4:**
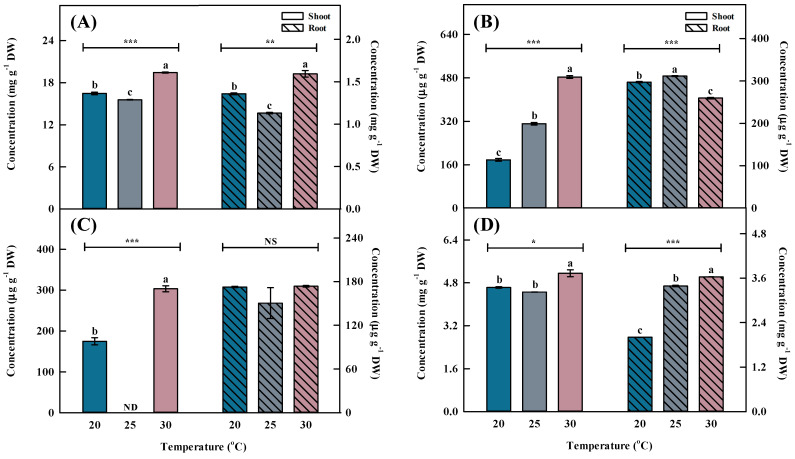
Individual phenolic acid concentrations by different temperatures for the shoots and roots of *Aster* × *chusanensis* (*n* = 8). (**A**) Chlorgenic acid, (**B**) p-hydroxybenzoic acid, (**C**) vanilic acid, and (**D**) benzoic acid. Bar height indicates the mean, and error bars show the standard deviation. Mean separation by Duncan’s multiple range test at 5% level. Different letters (a–c) above the bar mean significant differences (*, *p* < 0.05; **, *p* < 0.01; ***, *p* < 0.001). ND—non-detection; NS—no significant difference.

Temperature is one of the primary environmental factors that affect the accumulation of bioactive compounds in plants. Total phenolic acid concentration was highest (*p* < 0.001) at 30 °C, followed by 20 and 25 °C (Figure 3 and Figure 4). The total phenolic acid concentration was most affected by chlorogenic acids. A previous study reported that the phenolic acid concentrations were higher at 30 °C than at 25 °C (control) after inducing oxidative stress [26].

### 2.4. Phenolic Acid Concentrations in Aster × chusanensis Roots under Different Growth Temperatures

Similar to the shoot, the phenolic acid concentration in the root was highest (*p* < 0.001) at 30 °C, followed by 20 and 25 °C (Figure 3 and Figure 4). Chlorogenic acids were extracted at significantly higher (*p* < 0.01) concentrations from plants grown at 30 °C compared with the other growth temperatures. Furthermore, benzoic acid had the highest concentration among phenolic acids, showing a significant difference (*p* < 0.001) at 30 °C.

In plants, the level of responses to temperature stress varies depending on stress intensity [27,28]. Adaptive characteristics expressed in plants exposed to moderate stress intensity are called defense priming [29] and also occur during abiotic stress [30,31]. Excessive heat stress damages the photosystem and reduces plant production [32], and it changes the pattern of secondary metabolites such as the flavonoids and phenolic compounds as a defense mechanism [33]; however, moderate heat stress can repair stress damage by returning to normal temperature after stress treatment [34]. In the present study, 30 °C was not excessive but moderate heat stress because it did not harm growth, so it may represent both an adaptation process and a defense response such as secondary metabolite accumulation.

## 3. Materials and Methods

### 3.1. Plant Material and Growth Temperature Condition

**Figure 5:**
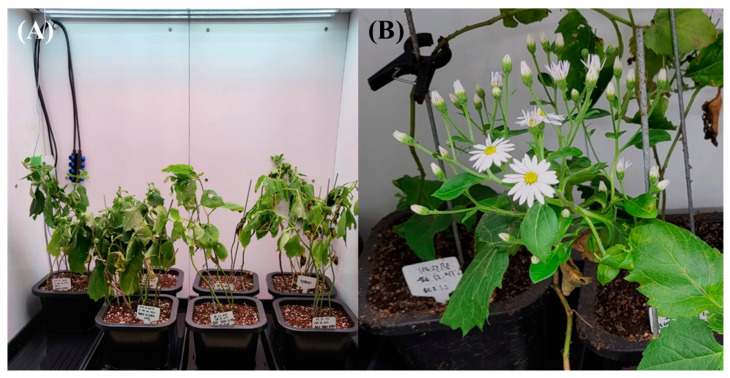
*Aster* × *chusanensis* grown in a growth chamber (**A**) and *Aster* × *chusanensis* used for the analysis of individual phenolic acid concentrations in each part (**B**).

*Aster* × *chusanensis* Y.S. Lim, Hyun, Y.D. Kim and H.C. Shin was collected on Ulleung Island, Ulleung-gun, Republic of Korea (37°31′44.4′′ N, 130°49′54.8′′ E and 37°32′031′′ N, 130°51′032′′ E). The plant materials were identified by the National Institute of Biological Resources. Growth chambers (Gaooze; KSTI, Seoul, Republic of Korea) were used to maintain the following conditions: temperature of 20 ± 3 °C, relative humidity of 70 ± 10%, light intensity of 140 ± 10 μmol m^−2^ s^−1^, and photoperiod of 12/12 h (day/night); the growth period was 6 days (Figure 5). Water was applied via bottom watering using distilled water when the soil surface had dried. Plant material was analyzed by separating the plants into leaves, stems, and roots.

**Figure 6:**
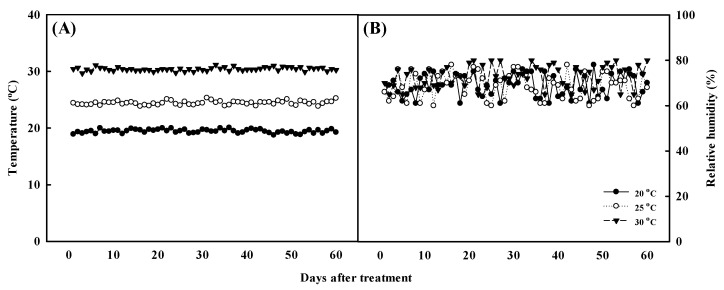
Temperature (**A**) and relative humidity (**B**) monitored at 2 h intervals during the experiment.

Using vegetative propagation, *Aster* × *chusanensis* individuals were separated from the mother plant, transplanted into a plastic pot containing horticultural soil (Tosilee, Yeast, Hapcheon-gun, Republic of Korea), and grown under different temperatures. Growth temperature conditions were 20 ± 2, 25 ± 2, and 30 ± 2 °C, as maintained using a growth chamber (VS-91G09M-4R, Visionbionex, Republic of Korea). Environmental conditions within the plant growth chamber were maintained at a relative humidity of 70 ± 10%, light intensity of 140 ± 10 μmol m^−2^ s^−1^, and a photoperiod of 12/12 h (day/night). Growth temperature and relative humidity were monitored at 2 h intervals during the experiment (Figure 6). After transplantation, the plants were irrigated for 1 week using distilled water and thereafter using Hoagland’s solution (electrical conductivity 1.2 dS m^−1^ and pH 6.0 at 24.2 °C) once per week. The plants were harvested after cultivation under these conditions for approximately 2 months.

### 3.2. Growth Characteristic Determination

Before conducting division cultivation and treating at different temperatures, plants were divided into leaves, stems, and roots. Then, the plant samples were dried in a dry oven (WOF-155, DAIHAN Scientific, Seoul, Republic of Korea) at 70 °C for 1 week and analyzed for phenolic acid concentrations. For growth temperature experiments, division cultivation was used. Plants grown at different temperatures for approximately 2 months were divided into shoots and roots, and fresh weight was recorded using an electronic scale (PAG214C, Ohaus Corporation, Parsippany, NJ, USA). Leaf area measurement was completed using Image J software, and each sample was subsequently dried in an oven at 70 °C for 3 days. The dry weight was then recorded, and the leaf was ground to powder for use in the next analysis.

### 3.3. Phenolic Acid Analysis

The phenolic acid concentrations were measured using a modified version of a previously described method [35]. The calibration curves of phenolic acids were set on nine points (1000, 500, 250, 100, 50, 10, 5, 1, and 0.5 µg/mL) of each standard. A stock solution of each compound was produced using dimethyl sulfoxide. The correlation coefficients of individual curves exceeded 0.998. The plant powder samples (1 g) were weighed using an electronic scale (PAG214C, Ohaus Corporation, Parsippany, NJ, USA) and placed in a conical tube. Then, 20 mL 50% HPLC-grade methyl alcohol diluted with HPLC-grade water was added to the powder followed by incubation (SH-800, SEYOUNG SCIENTIFIC) overnight at 25 °C. The extract was centrifuged (Centrifuge 5430R, Eppendorf, Hamburg, Germany) at 958× *g* for 10 min. The supernatant was filtered using a 0.45 µm membrane filter (PV2545, Chromdisc, Suwon, Republic of Korea), and 1 mL of extract was transferred to HPLC vials.

Phenolic acid concentrations were determined using HPLC (HPLC Agilent 1260 system, Agilent Technologies, Waldbronn, Germany) with an XTerra™ RP C18 column (4.6 mm × 250 mm, 5 μm; Waters, Milford, MA, USA). The injection volume of the sample was 20 µL, and the sample was analyzed in triplicate. The absorbance of phenolic acids was detected at 280 nm, and the column temperature was maintained at 30 °C. The flow rate was 1.0 mL min^−1^. As a mobile phase solvent, mobile phase A was 0.2% acetic acid in HPLC water, and mobile phase B was 0.2% acetic acid in acetonitrile. The elution gradient was applied as shown in Table 2.

We confirmed the limit of detection and the limit of quantification and then selected the individual phenolic acids (Table 3). Integration of peak and calculation of the quantification of individual phenolic acids were executed manually. The total phenolic acid concentrations were calculated by adding up the concentration of individual phenolic acids for each repetition and then deriving the average and standard deviation.

### 3.4. Chemicals

HPLC-grade reagents (water, acetonitrile [99.9%], and methyl alcohol [99.9%]) were purchased from Daejung Co. (Daejung Chemical & Materials Co., Ltd., Siheung, Gyeonggi-Do, Republic of Korea). The reference standards for phenolic acids (chlorogenic acid, p-hydroxybenzoic acid, vanillic acid, and benzoic acid) were purchased from Sigma-Aldrich (St. Louis, MO, USA).

### 3.5. Statistical Analysis

Analysis of secondary metabolites in each plant part was performed using six replicates per treatment. Analysis of growth characteristics and secondary metabolites by different temperatures was performed using five replicates. Differences in secondary metabolites at the various temperatures were examined using eight replicates. All data were calculated as the mean and standard deviation of each replication. Data analysis was performed using a one-way analysis of variance with the SAS program (SAS 9.4, SAS Institute, Cary, NC, USA). Duncan’s multiple range test was used to test differences among all treatments at *p* < 0.05.

## 4. Conclusions

Determining the phenolic acids in *Aster* plants, including in the leaves, stems, and roots, may provide useful information on *Aster* × *chusanensis*. Here, chlorogenic acid was the primary compound in *Aster* × *chusanensis* leaves. The various temperature conditions did not result in significant differences in the growth characteristics of *Aster* × *chusanensis* after the division propagation method. A growth temperature of 30 °C was efficient for phenolic acid biosynthesis. This study provides valuable information on the propagation methods and secondary metabolite concentrations under different temperatures of *Aster* × *chusanensis,* which is a natural hybrid species in South Korea.

## Figures and Tables

**Table 1:** Phenolic acid concentrations of each part of *Aster* × *chusanensis* (*n* = 6). Concentrations expressed in μg g^−1^ dry weight.

Phenolic Acid	Leaf	Stem	Root	Sig
Chlorgenic acid	28,215.82 ± 3711.68 ^z^ a ^y^	1593.63 ± 21.20 b	1164.48 ± 25.75 b	**
p-hydroxybenzoic acid	498.64 ± 1.33 a	155.10 ± 0.58 b	147.83 ± 0.79 c	***
Vanilic acid	354.12 ± 5.94 a	ND ^x^	207.26 ± 31.12 b	**
Benzoic acid	5231.75 ± 271.42 a	560.58 ± 0.67 c	1601.37 ± 60.51 b	***
Total	34,300.31 ± 3435.43 a	2309.29 ± 19.96 b	3120.91 ± 118.15 b	**

^z^ Mean ± standard deviation. ^y^ Mean separation within rows by Duncan’s multiple range test at 5% level. Different letters (a–c) indicate significant differences. Sig—significance; ** and *** mean *p* < 0.01 and *p* < 0.001, respectively; ND ^x^—non-detection.

**Table 2:** Elution gradient used for phenolic acid analysis.

Time (min)	Mobile Phase A (%)	Mobile Phase B (%)
0	100	0
3	97	3
5	95	5
8	90	10
10	85	15
15	95	5
17	92	8
19	90	10
20	85	15
22	80	20
26	85	15
27	80	20
28	70	30
32	60	40
36	40	60
37	70	30
38	60	40
40	50	50
45	40	60
55	20	80
60	10	90
65	0	100

**Table 3:** The LOQ and LOD of HPLC analysis.

Phenolic Acid	LOD (µg mL)	LOQ (µg mL)
	(3.3 × σ/S)	(10 × σ/S)
Chlorogenic acid	33.594	101.801
p-hydroxybenzoic acid	42.326	128.261
Vanillic acid	41.386	125.413
Benzoic acid	3.773	11.433

HPLC—high-performance liquid chromatography; LOD—limit of detection; LOQ—limit of quantification.

## Data Availability

All the data are available in the paper.
